# Prediction of Type 2 Diabetes Based on Machine Learning Algorithm

**DOI:** 10.3390/ijerph18063317

**Published:** 2021-03-23

**Authors:** Henock M. Deberneh, Intaek Kim

**Affiliations:** Department of Information and Communications Engineering, Myongji University, 116 Myongji-ro, Yongin, Gyeonggi 17058, Korea; henockmamo54@gmail.com

**Keywords:** type 2 diabetes, machine learning, prediction

## Abstract

Prediction of type 2 diabetes (T2D) occurrence allows a person at risk to take actions that can prevent onset or delay the progression of the disease. In this study, we developed a machine learning (ML) model to predict T2D occurrence in the following year (Y + 1) using variables in the current year (Y). The dataset for this study was collected at a private medical institute as electronic health records from 2013 to 2018. To construct the prediction model, key features were first selected using ANOVA tests, chi-squared tests, and recursive feature elimination methods. The resultant features were fasting plasma glucose (FPG), HbA1c, triglycerides, BMI, gamma-GTP, age, uric acid, sex, smoking, drinking, physical activity, and family history. We then employed logistic regression, random forest, support vector machine, XGBoost, and ensemble machine learning algorithms based on these variables to predict the outcome as normal (non-diabetic), prediabetes, or diabetes. Based on the experimental results, the performance of the prediction model proved to be reasonably good at forecasting the occurrence of T2D in the Korean population. The model can provide clinicians and patients with valuable predictive information on the likelihood of developing T2D. The cross-validation (CV) results showed that the ensemble models had a superior performance to that of the single models. The CV performance of the prediction models was improved by incorporating more medical history from the dataset.

## 1. Introduction

Diabetes is a chronic metabolic disorder that is identified by an abnormal blood glucose level, which is caused by either ineffective utilization or insufficient production of insulin [1]. The prevalence of diabetes in 2010 was estimated to be 285 million people worldwide (6.4% of adults). By 2030, that number is expected to rise to 552 million [2]. Based on the current growth rate of the disease, in 2040, one out of ten adults can be expected to have developed diabetes [3]. The prevalence of diabetes in South Korea has also increased dramatically; recent studies have shown that 13.7% of all South Korean adults have diabetes, and nearly a quarter have prediabetes [4].

Because those with diabetes often lack knowledge about the disease or are themselves asymptomatic, diabetes often remains undetected; nearly a third of diabetic patients are not aware of their status [5]. Uncontrolled diabetes results in serious long-term damage to several organs and body systems, including the kidneys, heart, nerves, blood vessels, and eyes [1]. Thus, advanced detection of the disease enables those at risk to take preventive action to inhibit the progression of the disease and improve quality of life [6].

To reduce diabetes’s effects and improve the quality of patient care, research has been conducted in several different sectors, including machine learning (ML) and artificial intelligence (AI) [3,7,8]. ML-based methods for diabetes occurrence prediction have been reported in multiple studies [3,9,10,11]. These methods are of two types: current condition identification (screening, diagnosis) and forward prediction approaches. Current condition identification methods deal with the classification of current data instances; forward prediction methods forecast the incidence of diabetes ahead of time using current and previous medical records [12].

In this study, we aim to develop a machine learning (ML) model to predict type 2 diabetes (T2D) occurrence in the following year (Y+1) using the feature values in the current year (Y). The prediction models group the input data instance into the specified condition: normal (non-diabetic), prediabetes, or diabetes. To build the prediction model, key features were first selected using a data-driven feature selection technique composed of an analysis of variance (ANOVA) test, a chi-squared test, and recursive feature elimination methods. We compared the performance of the prediction models—logistic regression (LR), support vector machine (SVM), random forest (RF), and XGBoost algorithms. We also utilized ensemble techniques such as a confusion matrix-based classifier integration approach (CIM), soft voting, and classifier stacking methods and compared the performance with the single models [13,14,15,16,17,18,19].

## 2. Background

### 2.1. Related Works

The availability of large electronic medical record collections compiled from multiple health facilities provides an opportunity within the current ML and AI trends to revolutionize diagnostic systems [12]. Despite some limitations in the reporting and interpretation of the performance of these approaches, their diagnostic capability resembles that of health-care professionals. Experts in these techniques can help clinicians understand what data is optimal for solving targeted problems, such as screening and forecasting tasks, and how and when that data can be obtained [12,20].

To facilitate early detection of T2D, numerous research studies employing ML techniques have been conducted. These studies include the development of screening, diagnosis, and prediction tools to detect the occurrence of the disease and the likelihood of its onset [5,21]. Screening methods for prediabetes using ML models for the South Korean population are presented in [5], which developed an intelligence-based screening model for prediabetes using a dataset from the Korean National Health and Nutrition Examination Survey (KNHANES) [22]. The KNHANES 2010 dataset, with 4685 instances, was used to train SVM and artificial neural network (ANN) based models, and the KNHANES 2011 dataset was used for validation. The authors claimed that the SVM model performed better than the ANN model, with an area under curve (AUC) value of 0.73. The study was limited to identifying a prediabetic condition only.

A model for predicting the onset of type 2 diabetes in non-diabetic patients with cardiovascular disease is presented in [21]. The study reported a T2D prediction model to forecast the occurrence of the disease within the follow-up period. The electronic health records (EHRs) for the study were collected from Korea University Guro Hospital (KUGH). The total number of features was 28, with 8454 subjects over five years of follow-up. The authors claimed that they had achieved a value of 78.0 in AUC measure for the logistic regression (LR) model. In this study, the dataset included only individuals with cardiovascular risks.

A comprehensive study on machine learning techniques for diabetes identification is presented in [23]. The study analyzed two essential data processors: PCA (Principal Component Analysis) and LDA (Linear Discriminant Analysis) for various machine learning algorithms. Through an experiment, they identified the best data preprocessor for each algorithm and conducted parameter tuning to find the optimum performance. Pima Indian data set was utilized to examine the performance of the algorithms. The highest accuracy obtained among the employed five algorithms (neural Network, Support Vector Machine, Decision tree, Logistic regression, and Naïve Bayes) was 77.86% using 10-fold cross-validation.

Machine learning algorithms also have been utilized to diagnose other types of chronic diseases. The study presented in [24] utilized ML algorithms to predict treatment success in a pediatric asthma cohort. The study predicted treatment outcomes in children with mild to severe asthma, based on changes in asthma control, lung function, and fractional exhaled nitric oxide (FENO)values after six months of controller medication use. The predictive possibilities were tested using the Random Forest (RF) and Adaptive Boosting (AdaBoost) machine learning algorithms. The results of this study will help to enable treatment optimization and implement the concept of precision medicine in pediatric asthma treatment.

### 2.2. Type 2 Diabetes (T2D)

Diabetes mellitus is a group of metabolic abnormality identified by hyperglycemia resulting from defects in insulin secretion, insulin action, or both [1]. According to the American Diabetes Association (ADA) guidelines, T2D is defined by fasting plasma glucose (FPG) levels above 125 mg/dL; the normal (non-diabetic) range is below 100 mg/dL [25]. It is highly affected by lifestyle activities, such as drinking, exercise, and dietary habits. T2D diminishes quality of life and lowers life expectancy. Several studies have shown that a combination of lifestyle improvement and medication intervention can prevent complications from the disease. Both early diagnosis and treatment of T2D are thus critical in preventing serious and potentially life-threatening complications in patients [21]. In this study, T2D was diagnosed according to the ADA guidelines. T2D is defined by FPG levels above 125 mg/dl; the normal range is below 100 mg/dL and between 100 and 125 mg/dL is considered prediabetes.

### 2.3. Feature Selection Techniques

Feature selection is the process of selecting a subset of the most relevant features in the dataset to describe the target variable. It improves computation time, generalization performance, and interpretational issues in ML problems [26,27]. Feature selection techniques are categorized as filter based, wrapper based, and embedded type. Filter-based techniques screen out features based on some specified criteria. Wrapper-based methods use a modeling algorithm that is taken as a black box to evaluate and rank features. The embedded methods have built-in feature selection approaches such as least absolute shrinkage and selection operator (Lasso) and random forest (RF) feature selection methods [28]. There are several types of feature selection techniques, including exhaustive search, Pearson correlation technique, chi-squared technique, recursive feature elimination, Lasso, and tree-based feature selection techniques. In this study, we used a data-driven feature extraction technique, which combines the ANOVA test, chi-squared test, and a tree-based recursive feature elimination technique.

#### 2.3.1. Analysis of Variance

Analysis of variance (ANOVA) is a well-known statistical method to determine whether there is a difference in means between two groups [29]. In this study, the ANOVA test was utilized to select the significant numerical features in predicting the occurrence of T2D. The ANOVA test uses the F statistic for feature ranking. The larger the value of the F statistic, the better the discriminative capacity of the feature [30]. The F value is calculated as:

(1) $$F=\left(\frac{SSB}{df_{b}}\right)/\left(\frac{SSW}{df_{w}}\right)$$

where SSB (sum of squares between groups) is the variation of group means from the total grand mean, and SSW (sum of squares within groups) is the sum of squared deviations from the group means and each observation. The degrees of freedom for mean square between and within is defined by $df_{b}\;$ and $df_{w}$, respectively [31]. For all numerical features in the dataset, the F value was calculated using Equation (1) and the features with the larger value were selected.

#### 2.3.2. Chi-Squared Test

The chi-squared test is a nonparametric statistical analyzing method. The technique calculates the chi-squared value using Equation (2) and selects the top *n* features [32]. In this work, the chi-squared test was employed to rank categorical features according to their significance in identifying the target class. Equation (2) is expressed as

(2) $$\chi^{2}=\overset{n}{\underset{i=1}{\sum }}\frac{{\left(x_{i}-E_{i}\right)}^{2}}{E_{i}}$$

where $x_{i}$ is observed frequencies, $E_{i}$ is expected frequencies, and n is the number of categories in the contingency table. For all categorical features in the dataset, the chi-squared value was calculated using Equation (1) and the features with the larger value were selected.

#### 2.3.3. Recursive Feature Elimination

Recursive feature elimination (RFE) is a recursive procedure to select features according to the accuracy of the model. The metric determines the discriminative capacity of features. At each iteration, the ranking score metric is calculated, and low-ranking features are eliminated. The recursive procedure is repeated until the desired number of features is achieved [33,34,35]. In this study, RFE was used as the final stage of the feature selection procedure. The detail of the feature selection procedure is presented in Section 3.2.

## 3. Methods

This section describes the methods used to develop a prediction model to forecast the occurrence of T2D in the following year. To generate the model, data preprocessing, feature selection, hyperparameter tuning, training, testing, and model evaluation procedures were performed.

### 3.1. Dataset

The dataset used in this research is a six-year electronic medical record collected from 2013 to 2018 at a private medical institute called Hanaro Medical foundation in Seoul, South Korea. It has 535,169 instances collected from 253,395 subjects and each instance has 1444 features. The subjects in the dataset were included in the dataset without any restrictions on occupation, sex, or gender. For privacy protection, the dataset does not contain any personal data, including subjects’ names and personal identification information. The average age of subjects is 41.2, with an age range of 18–108 and a sex ratio (males/females) of 1.25. The feature values in the dataset are a combination of the blood test (biochemical test), anthropometric measurements, and other diagnostic results. Also, It contains a questionnaire responded to by the patient at the hospital during the examination. Out of the total features, 140 of them were from the questionnaires. Subsequently, the dataset is the combination of numerical values from laboratory diagnostic results and categorical values from the questionnaire answers.

#### 3.1.1. Dataset Selection for the Experiment

The dataset contains records of subjects who visited the medical institute for from one to six years through the follow-up period. The total number of subjects used in this study was 253,395. Subjects who had at least two years of continuous annual medical check-ups during the follow-up period were selected as a target group for the experiment. We excluded subjects who had already been diagnosed with diabetes, hyperlipidemia, or hypertension, as well as those who took at least one medication for those diseases, because the dataset for the experiment required a transition from normal to three states.

#### 3.1.2. Missing-Data Handling

Data preprocessing is one of the significant steps in ML and data mining. It improves the quality of data and performance of ML models. The technique refers to cleaning and transforming the raw data to make it more suitable to train and evaluate prediction models. Data preprocessing includes data preparation, cleaning, feature selection, missing values handling, and transformation of data. The result expected after data preprocessing is a final dataset, that can be considered correct and useful for further data mining algorithms [36].

The collected EHRs were a high dimensional dataset. It is unlikely that all the features were obtained during the medical check because the required measurements were dependent on the subjects. To address the missing-values problem, several solutions were considered, including omission of the row with null values and replacing the missing values by mean, median, or mode values of the feature values [37]. Considering the large size of the dataset, records with null feature values were excluded from the dataset.

#### 3.1.3. Class Imbalance Problem

Most machine learning algorithms assume that the target classes share similar prior probabilities. However, in numerous real-world applications, this assumption is violated. When working with datasets that have a class imbalance, the machine learning classifier tends to be more biased towards the majority class, causing bad classification of the minority class. In such problems, most of the examples are labeled as one class, while fewer examples are labeled as the other class [38,39].

In our dataset normal-class instances accounted for 68.1% of the dataset, diabetes accounted for 4.3%, and prediabetes accounted for the remainder. The distribution of these three classes showed an imbalance, which could have resulted in poor prediction performance on the minority class for the prediction model [38]. To fix the problem, majority under-sampling and synthetic minority over-sampling (SMOTE) methods were utilized [40,41].

### 3.2. Data-Driven Feature Selection Procedure

This section presents a data-driven approach to select features for predicting T2D occurrence using statistical and ML methods. The dataset obtained through the above procedures contained both numerical variables from the diagnostic results and categorical entities from the questionnaire answers. The feature selection aimed to find a set of optimal features that could distinguish the three classes efficiently.

**Figure 1:**
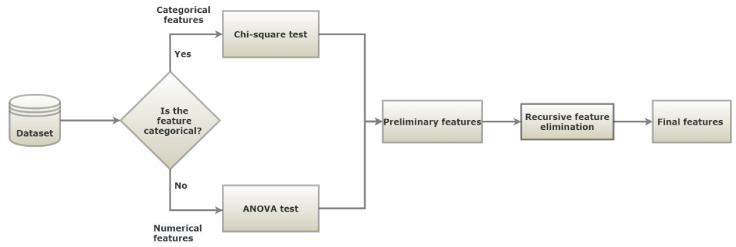
Feature selection procedure.

**Figure 2:**
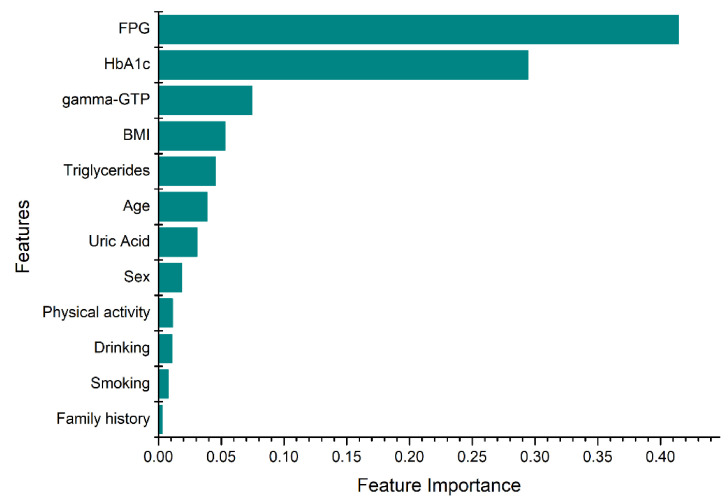
Feature importance ranking (FPG = fasting plasma glucose, HbA1c = hemoglobin A1c, BMI = body mass index, gamma-GTP = gamma glutamyl transpeptidase).

The feature selection procedure is shown in Figure 1. In the first step, the features set is split in two, based on the data types: numerical and categorical. Then, the appropriate metric was applied to rank the importance of the features. For numerical features, an ANOVA test was employed as a metric to select numerical features, while a chi-squared test was used for the categorical features. The selected features from both data types were combined, and the RFE technique was employed. RFE was conducted until the desired performance and number of features were achieved. On this technique, a tree-based approach was used to rank the features based on their level of importance. Finally, the most significant features were selected according to their importance, as shown in Figure 2. The selected features were fasting plasma glucose (FPG), body mass index (BMI), Gamma glutamyl transpeptidase (gamma-GTP), triglycerides, sex, age, uric acid, hemoglobin A1c (HbA1c), smoking, drinking, physical activity, and family history. Smoking status was divided into “currently smoking regularly”, “never smoked” and “had quit smoking”. Physical activity indicates the number of days the subject has engaged in physical exercise such as running, hillwalking, climbing stairs, jump roping for a minimum of 20 min. Family history with diabetes considers only parents and siblings diagnosed with T2D and drinking indicates the number of days the subject consumed alcoholic drinks.

The feature importance was computed as the node impurity which was weighted by the probability of reaching the node. The node probability was defined by the ratio of the number of samples that reach the node to the total number of samples [42]. The x-axis in Figure 2 indicates the normalized value of the feature importance. The higher the value the more important the feature. In general, the proposed data-driven feature selection method specified the most important and relevant features to indicate the occurrence of diabetes, and it is consistent with several studies [43,44,45,46,47,48,49,50].

### 3.3. Prediction Model

**Figure 3:**
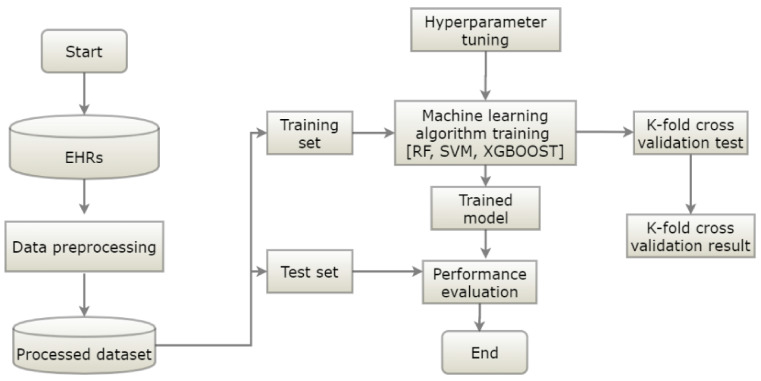
The architecture of the prediction model (RF = random forest, XGB = XGBoost, SVM = support vector machine).

This section explains the flow of the proposed diabetes occurrence prediction model. The proposed model had data preprocessing, training, and testing phases (Figure 3). The data preprocessing phase dealt with data cleaning and features selection. The preprocessed data was split into training and testing datasets. In the training phase, the prediction model was trained using the labeled training data, and hyperparameter tuning was applied to optimize the parameters of the model for better performance. To obtain the optimal parameters, we employed a tenfold cross-validated grid search on the tunable parameters of the models. First, we applied a general search with a wider range of parameters. Then, we applied a finer grid search in the neighborhood of the first selection to find the best values for the hyperparameters. The performance of the classifier was evaluated in the testing phase. RF, SVM, and XGBoost algorithms were utilized to generate prediction models.

Multiple classifiers are generated using a different combination of feature sets and aggregated to form the final predictor. Since the ensembled methods (CIM, ST, and SV) use all available classifiers information, their performance is better and/or more robust in most applications [51]. In this study, we utilized the classifier integration model with a confusion table [52], soft voting [18], and stacking classifier models [19].

Three sets of experiments were conducted to investigate the performance of the proposed prediction model. The first set of experiments dealt with the evaluation of the models using the test dataset and the ten-fold cross-validation (CV) technique. The CV technique randomly divided the dataset into ten subsets, and the experiments were conducted ten times iteratively. On each iteration, one of the ten subsets was used as test data, and the remaining nine subsets were used as a training set. The second set of experiments were performed to investigate the performance of the prediction model in comparison with the number of medical follow-up years used to train the prediction model. The training dataset for the experiments was generated by concatenating the medical records over the years. The number of years used to train the dataset ranged from two to four. The last set of experiments presented the cross-validation performance comparison between the selected 12-feature set and the well-known traditional predictors of T2D. The detailed results of the experiments are presented in Section 4.

## 4. Results

This section presents the experimental results of the proposed models. RF, SVM, and XGBoost algorithms were utilized to build the prediction models, and their performance was evaluated using the accuracy, precision, recall, and F1-score metrics.

### 4.1. Evaluation Metrics

Evaluation metrics were used to evaluate the model’s performance. In this study, we used accuracy, precision, recall, and F1-score for the metrics of the prediction. They represent how close the actual values and predicted values were, and each definition is shown in Table 1.

### 4.2. Model Performance

This work developed a prediction model to forecast the occurrence of T2D in the following year. The developed prediction model classified the input data instance as normal, prediabetes, or diabetes. This study utilized previous medical records to generate prediction models. The sizes of training and test datasets were 17,131 and 200, respectively, for each class.

To demonstrate the effectiveness of the prediction model, we conducted experiments using LR, RF, XGBoost, SVM, CIM, stacking classifier (ST), and soft voting (SV) algorithms. The base classifiers for the ensemble techniques (CIM, ST, and SV) were generated using different feature sets and the algorithms mentioned above. The comparative experimental results of the prediction models, in terms of accuracy, precision, recall, F1-score, Matthews Correlation Coefficient (MCC), and Cohen’s kappa score (KC) are presented in Table 2.

According to the experimental results, the performance difference among the single models (LR, RF, SVM, and XGBoost algorithms) was negligible. The best accuracy achieved for predicting the occurrence of diabetes was 73% on the test dataset, and the lowest was 71% from the LR model, which is considered as the existing statistical analysis approach. The confusion matrix of the RF model is presented in Table 3. As can be seen from the confusion matrix, the majority of the classification errors were from the prediabetes class. The derived precision values from the confusion matrix for the normal, prediabetes, and diabetes classes were 70%, 61%, and 90%, respectively. The lowest precision value was from the prediabetes class, which resulted in diminished overall precision. The difficulty of identifying the prediabetes class was a result of the overlap of the prediabetes class with the normal and diabetes classes. As shown in Table 3, the highest false positive instances in predicting both normal and diabetes classes were from prediabetes, with 58 and 16 instances, respectively. Furthermore, the model had the highest false positive instances from the prediabetes class. Thus, the high degree of class overlap between the classes was one of the main challenges that degraded the accuracy of the classifier.

**Figure 4:**
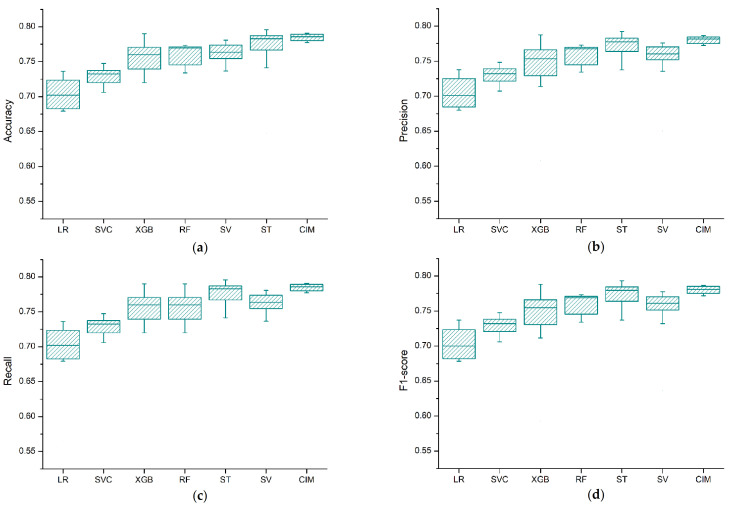
Box plot for the CV score of the prediction models (LR = logistic regression, RF = random forest, XGB = XGBoost, SVM = support vector machine, ST = stacking classifier, CIM = confusion matrix-based classifier integration approach): (**a**) accuracy, (**b**) precision, (**c**) recall, (**d**) F1-score.

To verify the cross-validation (CV) performance of the models, the experiments were conducted 10 times, and the average and the standard deviation of the accumulated accuracy, precision, recall, and F1-score values were used as evaluation metrics. Figure 4 depicts the box plot for the cross-validation results of the models. Based on the experimental results, we see clearly that the performance difference among the algorithms was negligible. However, the ensemble classifier approaches (CIM, ST, and SV) showed a performance improvement on the cross-validation results as compared to the single models.

**Figure 5:**
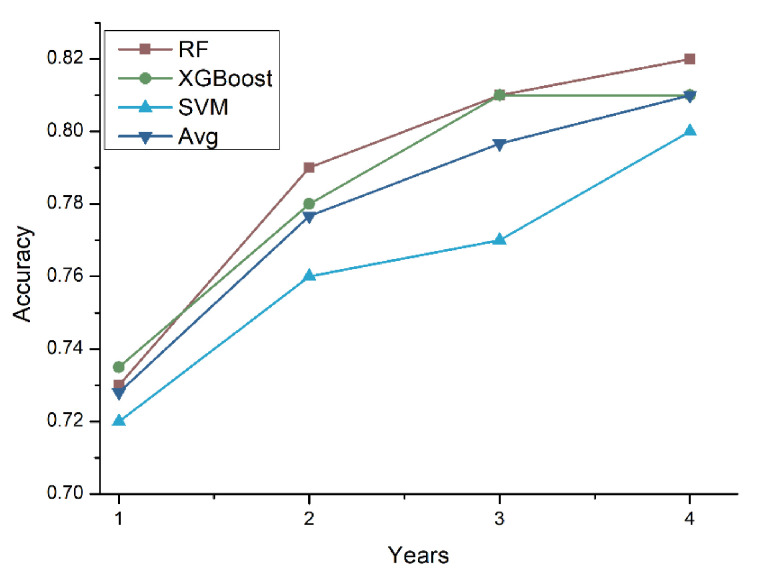
Accuracy comparison using a different number of years for training data (RF = random forest, XGB = XGBoost, SVM = support vector machine, Avg. = average).

To further investigate the accuracy of the prediction model with respect to the number of medical follow-up years, we conducted experiments by increasing the number of years used to train the prediction models. The number of years used to train the prediction model ranged from one year (Y) to four years (Y, Y-1, Y-2, Y-3). Figure 5 shows the tenfold cross-validation results. It is clear that as the number of years used to train the model increased, the accuracy of the prediction models also increased.

**Figure 6:**
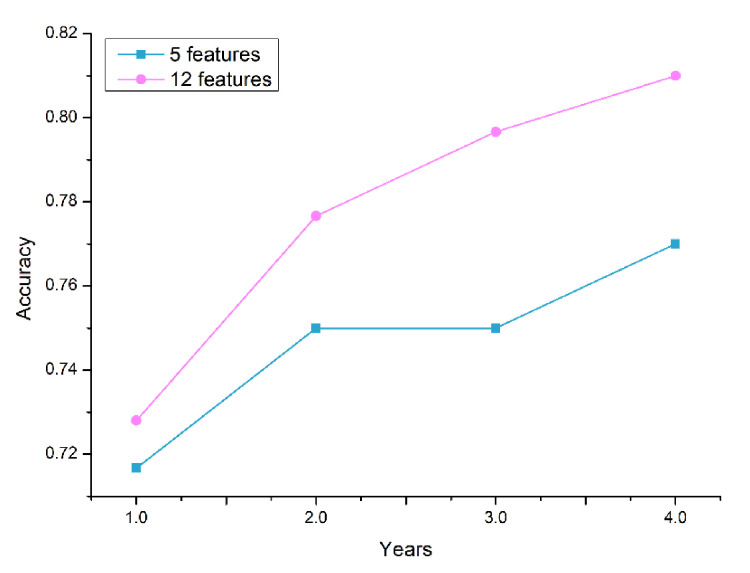
Accuracy comparison between the selected 12-feature set and the traditional predictors (5-feature set) using a different number of years for training data.

Figure 6 depicts the performance comparison between the selected 12-feature set and the well-known traditional predictors of T2D (5-feature set): FPG, HbA1c, BMI, age, and sex. The plot indicates the average accuracy comparison of the cross-validation results of the classifier models. Based on the experimental result, the accuracy of the models with the 12-feature set outperformed the traditional feature sets. The features added to the traditional predictors—triglycerides, gamma-GTP, uric acid, smoking, drinking, physical activity, and family history—improved the performance of the prediction models. Therefore, in addition to the traditional predictors of T2D, clinicians should pay attention to the changes in gamma-GTP, uric acid, and triglycerides over the years.

## 5. Discussion

This study proposed a machine learning model to predict the occurrence of T2D in the following year. While previous works in [21] and [53] developed a scheme for forecasting the occurrence of diabetes, this paper dealt with the possible transition among three classes: normal, prediabetes, and diabetes. Few studies have addressed the prediction of prediabetes, as most research has been focused on the prediction of undiagnosed diabetes.

In this study, a large dataset and ensemble ML techniques were employed to develop the prediction models as compared to the studies mentioned above. Furthermore, the impact of the cumulated medical data on the prediction accuracy was also presented by changing the number of years used to train the models. A data-driven feature selection was employed to find predictors that were significant for detecting the distinct classes in the dataset. The resultant 12 features were FPG, HbA1c, triglycerides, BMI, gamma-GTP, age, uric acid, sex, smoking, drinking, physical activity, and family history. FPG and HbA1c were the most important predictors based on the information-gain criteria; they were followed by gamma-GTP, BMI, triglycerides, and age. Compared to using the traditional five predictors of T2D (FPG, HbA1c, BMI, age, and sex), the proposed models employing the selected features showed a superior prediction performance. When four years of data were utilized in training, the maximum CV accuracy was 81% for the selected features and 77% for the traditional features. It can be concluded that the additional seven features contributed to improved accuracy of prediction. We also note that in addition to the traditional predictors, clinicians must pay attention to the changes in gamma-GTP, uric acid, and triglycerides over the years.

The study presented in [5] reported the application of an ML model to identify the occurrence of prediabetes in advance. In their study, they have indicated the difficulties of predicting the prediabetes condition. The best accuracy presented was 69.9% for the KNHANES dataset. Our experimental results have shown a better prediction performance in predicting the occurrence of not only diabetes and normal but also the prediabetes condition too. The highest CV classification accuracy observed was 78% by using last year’s medical records as training data. However, the performance of the prediction model was improved by increasing the number of years to train the models. The study presented in [53] reported a comparison of three data mining models for predicting diabetes or prediabetes by risk factors. The dataset for the study was collected from two communities in Guangzhou, China: 735 patients confirmed to have diabetes or prediabetes and 752 normal controls. The risk factors (predictors) used were age, family history of diabetes, marital status, education level, work stress, duration of sleep, physical activity, preference for salty food, gender, eating fish, drinking coffee, and body mass index. Three ML algorithms: logistic regression, artificial neural networks (ANNs), and decision tree models were employed for predicting diabetes or prediabetes using the predictors. The decision tree model (C5.0) had the best classification accuracy (77.87%), followed by the logistic regression model (76.13%), and the ANN gave the lowest accuracy (73.23%).

LR, RF, SVM, XGBoost, CIM, stacking classifier, and soft voting algorithms were utilized to generate the prediction models. Experimental results showed that the generated prediction models performed slightly better than the LR model, the existing statistical analysis method. However, the performance difference among the algorithms was negligible on the test data. This can be explained by class overlap in the feature space. The prediabetes class especially had a high degree of class overlap with normal and diabetes classes. The confusion matrix results confirmed that most of the prediction errors were from the prediabetes class. This lowered the overall performance of the prediction models and limited the maximum accuracy to 73%.

The CV results showed a significant performance difference among the prediction models. The ensemble models (CIM, ST, and SV) had a superior CV performance to that of the single models including LR. The CV performance of the prediction models was improved by incorporating more medical history from the dataset. Overall, the results of the present study demonstrated that the generated prediction models performed better than the existing clinical screening model (LR). The application of the developed prediction models and findings of this study redound to the benefit of both clinicians and patients. The models can be used as viable support in clinical decision-making and patient counseling for practitioners. Furthermore, early prediction of the disease enables diabetes patients and those at risk for diabetes to take preventive measures that can delay the progression of the disease and its life-threatening complications.

This study has certain limitations. First, FPG level was the only measurement that was used to define normal, prediabetes, and diabetes; HbA1c and oral glucose tolerance test (OGTT) were not taken into consideration. However, the use of FPG level was consistent with the model developed by [5,54]. Second, in this study 10-fold cross-validation was utilized in the evaluation of the models. However, the development and validation of the models were conducted with only one dataset. Thus, it is compulsory to utilize additional data sources to verify the models derived in this study.

Our study suggests two additional investigations that are worth pursuing. The first would be to incorporate diverse datasets to mitigate the difficulty of classifying prediabetes, which stems from the overlap with normal and diabetes classes. The second would be to increase the accessibility of the prediction models and improve the user experience for web and mobile applications.

## 6. Conclusions

In this paper, we proposed a T2D occurrence prediction model that can forecast the occurrence of T2D in the following year (Y + 1) as normal, prediabetes, or diabetes. LR, RF, XGBoost, SVM, and ensemble classifiers (CIM, ST, and SV) were utilized to generate the prediction models. Feature selection was employed to select the most significant features that can distinguish the three classes efficiently. The selected features were FPG, HbA1c, triglycerides, BMI, gamma-GTP, gender, age, uric acid, smoking, drinking, physical activity, and family history. Experimental results showed that the performance of the generated prediction model was reasonably good at forecasting the incidence of T2D in the Korean population. The model can provide both clinicians and patients with valuable information on the incidence of T2D ahead of time, which would help patients take measures to mitigate T2D risk, progression, and related complications. Furthermore, it can be used as a viable support in clinical decision-making for practitioners and diabetes educators to improve the quality of life of patients.

## Figures and Tables

**Table 1:** Evaluation metrics.

Metric	Definition
Accuracy	$=\frac{TP+TN}{TP+FP+FN+TN}$
Precision	$=\;\frac{TP}{TP+FP}$
Recall	$=\;\frac{TP}{TP+FN}$
F1-score	$=\;\frac{2*\left(\;recall*precision\right)}{recall+precision}$

TP = true positive, TN = true negative, FP = false positive, FN = false negative.

**Table 2:** Performance comparison of the generated prediction models on the test dataset

	Accuracy	Precision	Recall	F1-score	MCC	KC
LR	0.71	0.71	0.71	0.71	0.56	0.56
RF	0.73	0.74	0.73	0.74	0.60	0.60
XGBoost	0.72	0.74	0.72	0.73	0.58	0.58
SVM	0.73	0.74	0.74	0.74	0.60	0.60
CIM	0.73	0.73	0.73	0.73	0.59	0.59
Stacking classifier	0.72	0.75	0.72	0.73	0.58	0.58
Soft voting	0.73	0.74	0.73	0.73	0.59	0.59

LR = logistic regression, RF = random forest, SVM = support vector machine, CIM = confusion matrix-based classifier integration approach, MCC = Matthews Correlation Coefficient, and KC = Cohen’s kappa score.

**Table 3:** Confusion matrix of the RF model.

	Normal	Prediabetes	Diabetes
Normal	148	58	4
Prediabetes	51	126	29
Diabetes	1	16	167

## Data Availability

The data presented in this study are available on reasonable request from the corresponding author. The data are not publicly available due to ethical requirements.
